# The Effect of Parental Social Integration on the Physical Examination Utilization for Young Migrant Children: A National Cross-Sectional Study in China

**DOI:** 10.3389/fpubh.2021.755726

**Published:** 2022-01-12

**Authors:** Zhengyue Jing, Shiya Zhang, Nan Zhang, Mei Sun, Chengchao Zhou

**Affiliations:** ^1^School of Health Policy and Management, Nanjing Medical University, Nanjing, China; ^2^Centre for Health Management and Policy, School of Public Health, Cheeloo College of Medicine, Shandong University, Jinan, China; ^3^Department of Health Policy and Management, School of Public Health, Fudan University, Shanghai, China; ^4^Social Statistics, Manchester Institute for Collaborative Research on Ageing, University of Manchester, Manchester, United Kingdom; ^5^National Health Commission of the PRC (NHC) Key Laboratory of Health Technology Assessment, Fudan University, Shanghai, China; ^6^National Health Commission of the PRC (NHC) Key Lab of Health Economics and Policy Research, Shandong University, Jinan, China

**Keywords:** social integration, social factors, child health services, parent-child relations, transients and migrants

## Abstract

**Purpose:** Physical examination is a key component of child health management. Migrant children are a vulnerable group with lower healthcare service utilization, and this study aims to explore the effect of parental social integration on the physical examination service utilization for young migrant children under 6 years old in China.

**Method:** This study conducted a secondary data analysis of the 2014 National Internal Migrant Dynamic Monitoring Survey in China. A total of 2,620 participants were included in this study. A total of 22 indicators were selected to measure social integration. Multivariate logistic regression was used to explore the association between parental social integration and physical examination use of young migrant children.

**Results:** More than half (66.4%) of the migrant children aged 0–6 years had used free physical examination. Parental social integration, especially structural integration, was associated with the physical examination utilization of migrant children. Specifically, those migrant children's parents who had medical insurance (*P* < 0.05; OR = 1.29), who had participated in local activities (*P* < 0.001; OR = 1.98), who had registered local residents as neighbors (*P* < 0.05; OR = 1.34), and who had a deep sense of self-identity (*P* < 0.05; OR = 1.09) were more likely to take children to use physical examination.

**Conclusions:** This study provided evidence that parental social integration was associated with migrant children's physical examination utilization, and this association was multifaceted, lying in the dimensions of economic, structural, and psychological integration. Improving the social integration of migrant parents would be effective to enhance the migrant children's healthcare service utilization.

## Introduction

With the rapid development in China, the social-economic disparity across regions has caused a considerable amount of the internal migrant population. The migrant population was approximately 286 million in 2020, accounting for 20.3% of China's total population (1). Recently, more and more migrant parents bring their children together with them to the sites where they work, leading to an increasing new group called “migrant children”. In 2015, there were about 34.26 million migrant children aged 0–17 years, accounting for 12.9% of the total number of children in China (2). Correspondingly, the number of migrant children aged 0–5 years has been continually on the rise, from 6.64 million in 2000 to 10.53 million in 2015 (3).

Under the current household registration (Hukou thereafter) system, the migrants have limited access to social welfare, including housing, employment, health care, and public health services (4). Migrant children being a vulnerable group, are more likely to face higher health risks and have limited access to health care services than non-migrant children (5, 6). The New York Declaration of the United Nations General Assembly urged all countries to protect the human rights and freedoms of migrant children and to have access to basic services (7, 8). To improve the accessibility to health care services for migrant children, China launched health management for migrant children aged 0–6 years in 2013 (9). Physical examination is a key component of child health management, which is provided free of charge to children in each period, enabling early detection, diagnosis, and treatment of children's disease, ultimately reducing the occurrence of diseases and improving the quality of life of children.

Nevertheless, migrant children's health service utilization was associated with parental characteristics. A study found that parental birthplace affected access to preventive health services of children and adolescents in the United States (10). A review indicated that the Expanded Program on Immunization's vaccination of migrant children relied on the parental decision (11). However, previous studies mainly focused on the influence of parents' socio-demographic characteristics (such as educational attainment, socioeconomic status) on the healthcare services use among the migrant children (12), few studies have explored whether parental social integration affects health care service utilization of their migrant children. Social integration refers to the process of integrating newcomers into the social structure of the host regions. It is one of the most important factors affecting the health status and health service utilization of migrants, and also can be attributed to the root cause of many related problems in the process of migration (13, 14).

Therefore, this study aims to explore the effect of parental social integration on the physical examination utilization of young migrant children in China. To do so, this study has the following objectives. First, assess the status of the physical examination for young migrant children. Second, study the association between parental social integration and physical examination utilization among young migrant children.

## Methods

### Data Source and Sample

This study conducted a secondary data analysis of the 2014 National Internal Migrant Dynamic Monitoring Survey (NIMDMS) (15). The survey has been funded and organized by the Nationally Population and Family Planning Commission of the People's Republic of China since 2009 (NPFPC). The data in 2014 was chosen because it was the most comprehensive thematic survey on the social integration of the migrant population in China. The 2014 NIMDMS collected data from a nationally representative sample of Chinese migrants aged 15–59 years old who did not have local Hukou and had settled in the host cities for more than 1 month, selected using stratified three-stage probability proportionate to size sampling.

For this study, we included data from respondents whose children were born after June 2007 and settled in the local city for more than 1 year. In addition, if the respondents had more than one migrant child who met the inclusion criteria, the youngest one was selected. Finally, a total of 2,620 respondents were included in the analysis.

### Measurement

1. Dependent variables

The utilization of physical examination among migrant children was measured by the respondent's self-reported question: “Have your children had received a free health physical examination in the past 12 months?”. If the answer was “No”, the utilization of physical examination was coded as “no”, otherwise it was coded as “yes”.

2. Social integration

Based on the existing social integration indicator systems proposed by Zhou and Yang (16, 17), combined with the questionnaire used in the survey, this study measured through four dimensions: economic integration, structural integration, cultural integration, and psychological integration. Among them, economic integration was measured by three indicators: average monthly household income, occupation, and medical insurance; structural integration was measured by two indicators: activity participation and types of neighbors. The indicators of cultural integration and psychological integration were extracted by principal component factor analysis (See Supplementary Table 1). Cultural integration was measured by indicators 1–4, with a total score ranging from 5 to 20 points, the higher score indicated the worse integration. Psychological integration was measured by contact intention (indicators 5–9), self-identity (indicators 10–14), and self-perception (indicators 15–17), where the total scores of contact intention and self-identity ranged from 5 to 20 points, with higher scores indicated better integration, and the total scores of self-perception ranging from 3 to 12 points, with higher scores indicated worse integration.

3. Statistical analysis

This study used IBM SPSS version 22 (IBM Corporation, Armonk, NY, USA) to conduct the statistical analysis. First, descriptive analyses were performed to describe the social-demographic characteristics of the respondent and their migrant children. Second, the principal component factor analysis was adopted to extract common factors and to compute the scores for dimensions of social integration. Finally, two multivariate logistic regression models were employed to explore the association between parental social integration and the utilization of physical examination of migrant children using odds ratio (OR) and 95% CIs. Sampling weights were used in all the analyses to adjust for the design effect (18, 19).

## Results

### The Socio-Demographic Characteristics

The characteristics of the respondent and their children were presented in Table 1. About 66.4% of migrant children had used free physical examination in the past year. Among the 2,620 respondents, most of them were male (53.5%), were more than 30 years old (60.2%), with the education level of junior school or below (59.4%), were rural origin (85.3%), were married (98%), were intra-provincial migrants (52.3%), and had been migrants <5 years (59.2%).

**Table 1:** Socio-demographic characteristics of migrant children and their parents in China, 2014.

**Characteristics**	**Frequency (%)**	**Physical examination**		***P*-value**
		**Yes**	**No**	
**Total**	2,620 (100.0)	1,740 (66.4)	880 (33.6)	
**Children's characteristics**				
**Gender**				0.271
Boy	1,527 (58.3)	1,001 (65.5)	526 (34.5)	
Girl	1,093 (41.7)	739 (67.6)	354 (32.4)	
**Age (years)**				<0.001
≤ 3	1,366 (52.1)	957 (70.1)	409 (29.9)	
3-6	1,254 (47.9)	783 (62.4)	471 (37.6)	
**Duration in inflow areas (years)**			0.304	
≤ 3	1,891 (72.2)	1,267 (67.0)	624 (33.0)	
>3	729 (27.8)	473 (64.9)	256 (35.1)	
**One child**				0.020
Yes	1,515 (57.8)	1,034 (68.2)	481 (31.8)	
No	1,105 (42.2)	706 (63.9)	399 (36.1)	
**Parental characteristics**				
**Gender**				0.187
Male	1,402 (53.5)	947 (67.5)	455 (32.5)	
Female	1,218 (46.5)	793 (65.1)	425 (34.9)	
**Age (years)**				0.205
<30	1,042 (39.8)	707 (67.9)	335 (32.1)	
≥30	1,578 (60.2)	1,033 (65.5)	545 (34.5)	
**Educational attainment**				0.038
Primary education or below	143 (5.5)	83 (58.1)	60 (41.9)	
Junior education	1,412 (53.9)	929 (65.8)	483 (34.2)	
Senior education or above	1,065 (40.6)	728 (68.4)	337 (31.6)	
**Hukou**				0.140
Rural	2,234 (85.3)	1,471 (65.8)	763 (34.2)	
Urban	386 (14.7)	269 (70.0)	117 (30.0)	
**Marital status**				0.217
Single	53 (2.0)	31 (58.5)	22 (41.5)	
Married	2,567 (98.0)	1,709 (66.6)	858 (33.4)	
**Movement area**				0.001
Inter-provincial	1,370 (52.3)	871 (63.6)	499 (36.4)	
Intra-provincial	1,250 (47.7)	869 (69.5)	381 (30.5)	
**Duration in inflow area (years)**			0.006	
1-	1,552 (59.2)	998 (64.3)	554 (35.7)	
5-	1,068 (40.8)	742 (69.5)	326 (30.5)	

### Social Integration

Social integration was measured by economic, cultural, structural, psychological (Table 2). Regarding economic integration, 33.3% were in quartile 1(Q1), about 87.3%were employed, and 87.5% had medical insurance in their host cities. In the aspect of structural integration, 64.5% did not participate in local activities, and 43.1% of neighbors were non-registered residents. As for cultural integration, the average score of the migrant parents was 14.67. Regarding the psychological integration, the average score of contact intention, self-identity, and self-perception was 17.71, 16.52, and 5.47, respectively. The migrant children's physical examination utilization differed significantly by medical insurance (*P* = 0.002), participation in activities (*P* < 0.001) and types of neighbors (*P* = 0.003).

**Table 2:** Social integration of migrant parents in China, 2014.

**Characteristics**	**Frequency (%)**	**Physical examination**		***P*-value**
		**Yes**	**No**	
**Economy integration**				
**Monthly average household income** ^**a**^				0.123
Q1	871 (33.3)	560 (64.3)	311 (35.7)	
Q2	453 (17.3)	297 (65.6)	156 (34.4)	
Q3	601 (22.9)	397 (66.1)	204 (23.9)	
Q4	695 (26.5)	486 (69.9)	209 (30.1)	
**Occupation**				0.916
Unemployed	333 (12.7)	222 (66.7)	111 (33.3)	
Employed	2,287 (87.3)	1,518 (66.4)	769 (33.6)	
**Medical insurance**				0.002
Yes	2,293 (87.5)	1,548 (67.5)	745 (32.5)	
No	327 (12.5)	192 (58.7)	135 (41.3)	
**Structure integration**				
**Participate in activities**				<0.001
Yes	931 (35.5)	709 (76.2)	222 (23.8)	
No	1,689 (64.5)	1,031 (61.0)	658 (38.9)	
**Types of neighbors**				0.003
Migrants	1,130 (43.1)	725 (64.2)	405 (35.8)	
Local residents	568 (21.7)	410 (72.2)	158 (27.8)	
Same^b^	922 (35.2)	605 (65.6)	317 (34.4)	
**Culture integration** ^**c**^	14.67 ± 2.85	14.69 ± 2.89	14.64 ± 2.78	0.689
**Psychological integration** ^**c**^				
Contact intention	17.71 ± 2.57	17.77 ± 2.59	17.59 ± 2.53	0.091
Self-identity	16.52 ± 2.79	16.65 ± 2.81	16.26 ± 2.75	0.840
Self-perception	5.47 ± 1.87	5.53 ± 1.79	5.44 ± 1.90	0.149

a *Quartile 1 (Q1) is the poorest and Quartile 4 (Q4) is the richest*.

b *The number of registered residents and non-registered residents is the same*.

c *Culture and psychological integration were presented as Mean ± SD and analyzed by using a t-test*.

### Association Between Parental Social Integration and Migrant Children's Physical Examination Utilization

Table 3 shows the association between parental social integration and migrant children's physical examination utilization. The unadjusted model 1 only included the parental social integration, including dimensions of economic integration, structural integration, and psychological, which were statistically associated with the migrant children's physical examination utilization. After adjusting for the potential confounding factors including children's age, only one child, parents' education degree, movement area, duration in inflow area, model 2 found parental social integration, including dimensions of economic integration, structural integration, psychological, were still statistically associated with the migrant children's physical examination utilization. Those migrant children whose parents had medical insurance (*P* = 0.042; OR = 1.29), had participated in local activities (*P* < 0.001; OR = 1.98), had registered local residents as neighbors (*P* = 0.014; OR = 1.34), and had a deep sense of self-identity (*P* = 0.03; OR = 1.09) were more likely to use physical examination service. Besides, some other factors were also found to be associated with the migrant children's physical examination utilization. Those migrant children who aged over 3 years (*P* < 0.001; OR = 0.64), and were the only child in the family (*P* = 0.025; OR = 0.82) were less likely to use physical examination, and those parents who were inter-provincial migrants (*P* = 0.015; OR = 1.24) and had been migrants more than 5 years (*P* < 0.001; OR = 1.37) were more likely to take their children to use physical examination service.

**Table 3:** The relationship between parental social integration and their migrant children's physical examination utilization in China, 2014.

**Variables**	**Model 1 (Unadjusted)**			**Model 2 (Fully adjusted)**		
	***P*-value**	**OR**	**OR 95%CI**	***P*-value**	**OR**	**OR 95%CI**
**Economy integration**						
**Monthly average household income** ^**a**^						
Q1		1.0			1.0	
Q2	0.790	1.03	0.81–1.32	0.616	1.06	0.83–1.36
Q3	0.644	1.05	0.84–1.32	0.567	1.07	0.85–1.35
Q4	0.060	1.26	0.99–1.54	0.051	1.26	0.99–1.58
**Occupation**						
Unemployed		1.0			1.0	
Employed	0.342	0.88	0.69–1.14	0.522	0.92	0.71–1.19
**Medical insurance**						
No		1.0			1.0	
Yes	**0.024**	1.33	1.04–1.69	**0.042**	1.29	1.00–1.66
**Structure integration**						
**Participate in activities**						
No		1.0			1.0	
Yes	**<0.001**	1.97	1.64–2.36	**<0.001**	1.98	1.64–2.38
**Type of neighborhood**						
Migrants		1.0			1.0	
Local residents	**0.004**	1.39	1.11–1.75	**0.014**	1.34	1.06–1.69
Same^b^	0.675	1.04	0.86–1.25	0.770	1.03	0.85–1.24
**Culture integration**	0.203	0.95	0.88–1.03	0.123	0.93	0.86–1.01
**Psychological integration**						
Contact intention	0.936	1.00	0.92–1.09	0.956	0.99	0.92–1.08
Self-identity	**0.012**	1.11	1.02–1.21	**0.030**	1.09	1.00–1.19
Self-perception	0.888	1.00	0.93–1.09	0.657	1.02	0.94–1.11
**Children's characteristics**						
**Age (Years)**						
**≤3**					1.0	
**>3**				**<0.001**	0.64	0.54–0.76
**One child**						
Yes					1.0	
No				**0.025**	0.82	0.68–0.97
**Parental characteristics**						
**Educational attainment**						
Primary education or below					1.0	
Junior education				0.178	1.29	0.89–1.86
Senior education or above				0.227	1.26	0.86–1.85
**Movement area**						
Inter-provincial					1.0	
Intra-provincial				**0.015**	1.24	1.04–1.48
**Duration in inflow area (Years)**						
1-					1.0	
5-				**<0.001**	1.37	1.15–1.64

*The P-values in boldface mean statistical significance at 5% level*.

a *Quartile 1 (Q1) is the poorest and Quartile 4 (Q4) is the richest*.

b *The number of registered residents and non-registered residents is the same*.

*Adjusted for age, one child, parents' educational attainment, movement area, and duration in the inflow area*.

## Discussion

This study found that 66.4% of the migrant children aged 0–6 years had used free physical examination services. The utilization rate of physical examination services in this study was lower than the 83.5% among the migrant children aged under 7 years in Zhejiang province in 2013 (20). It was higher than the 41.1% of migrant children under 6 years old in Xinjiang province in 2015 (21). There were some disparities in the utilization rate of physical examination services among migrant children across different provinces in China, which may be related to the level of economic development in different provinces. In addition, even though provided freely, there were still over 30% of the migrant children did not use physical examination services in the inflow areas, which should be studied profoundly in the follow-up research, so as to identify the main determinants of the non-use of this service, and to improve the utilization rate among the migrant children aged 0 to 6 years.

This study provides evidence that parental social integration was associated with migrant children's physical examination utilization, and this association was multifaceted, lying in the dimensions of economic, structural, and psychological integration. Specifically, concerning economic integration, parental medical insurance status was found to be a determinant for migrant children's physical examination service use. Medical insurance is an effective tool to promote access to healthcare services (22). A review found that one of the most important barriers for labor-migrant to access health services was the lack of health insurance (8). One possible explanation for this finding might be that insured parents have strong social security awareness, tend to use physical examination services to find potential health risks for their children so that effective intervention can be developed promptly.

Structural integration, which was measured by activity participation and type of neighbors, was found to be positively associated with migrant children's physical examination utilization. At present, the migrant population was still in a relatively isolated state and trying to integrate into mainstream society. A study conducted in Ethiopia showed that the migrant status of women had a negative impact on child immunization use, and it was largely due to their limited social network and the disconnection of their host community (23). Participation in social activities was one of the crucial ways for migrants to obtain information (such as healthcare information), resources, and opportunities, which could create a beneficial environment for migrants to expand their social network and foster a sense of belonging. In addition, it was beneficial to maintain their physical and mental health (24, 25). As a result, migrant parents who participated in activities were more willing to integrate into the inflow cities, and more likely to use local health services.

Migrant parents with local registered residents as neighbors were more likely to use physical examination services. Hou et al. (26) found that migrants living in the community with a higher composition of local residents had a higher probability of using public health services, which was consistent with the finding of the current study. Localized social interaction could help migrants to be better familiar with the local culture, customers, decrease exclusion of mainstream social settings and have a strong sense of belonging to the inflow areas. On the other hand, migrants could obtain local information from their neighbors, including information on how to use the local health services (16, 27). A study had suggested that mothers who were socially integrated can obtain relevant oral health information from their surroundings individuals, which indirectly improves children's oral health service utilization (28).

In the aspect of psychological integration, only the migrant parents with a deep sense of self-identity were of higher probability to take their migrant children to use physical examination. The migrants are often accompanied by the term “self-identity”, which was an important indicator of the integration of the migrants into mainstream society. It refers to mutual recognition and acceptance between the migrant population and the local residents in the process of two-way social interaction. A study found that migrant parents in the middle-acculturation were less likely to take their children to use health services than those in the high-acculturation, largely because they perceived more personal and social discrimination (29). Accumulated evidence has shown that higher identity integration was positively associated with lower levels of stressors, higher social adjustment, and social support (30–33). Migrant parents with a deep sense of self-identity were more likely to have less psychological pressure and a stronger sense of identity and were willing to actively adapt to the environment in inflow areas. Therefore, they tended to regard themselves as local residents and were more willing to take their children to utilize the local health services, including physical examination services.

Furthermore, this study found parents' movement area and duration in the inflow areas, children's age, and whether as an only child in the family were the determinants for physical examination utilization. Regarding the parental characterizes, parents who were from intra-provincial migrant status and have lived in their local area for more than 5 years were more likely to take children to use physical examination. The customs and living habits of the place of origin were more similar to those of the new residence for intra-provincial migrants, and the longer stayed in the new residence, the more familiar they were with that place (34). About the characteristics of migrant children, children aged over 3 years and who were not only one child in the family were less likely to use physical examination services. This finding may be partly because the younger children and only children received more attention and care in the family under the traditional Chinese culture atmosphere, thus their parents were more likely to take them to use physical examination service.

This study had some limitations. First, the cross-sectional survey cannot predict the causal relationship between parental social integration and migrant children's physical examination utilization. Second, although this study used many indicators to measure social integration, due to the limitations of existing data, it still did not include other potential factors that may affect the utilization of physical examination, such as language, social support. Third, the physical examination utilization was self-reported, which may be over-reported or under-reported due to recall bias. Finally, this study only investigated Chinese migrants, so the main findings of this study should be interpreted with caution, especially for inter-country/continental migrants, as the language, tradition, and culture largely differed across countries.

## Conclusion

This study provided evidence that parental social integration was associated with migrant children's physical examination utilization. This association was multifaceted, lying in the dimensions of economic, structural, and psychological integration. The government should take measures to improve the social integration of migrant parents, so as to enhance the migrant children's healthcare utilization in the inflow areas.

## Data Availability Statement

The raw data supporting the conclusions of this article will be made available by the authors, without undue reservation.

## Author Contributions

CZ and MS contributed to the conception and design of the study. ZJ and SZ performed the statistical analysis and wrote the first draft of the manuscript. NZ, MS, and CZ reviewed and edited the manuscript, were responsible for visualization, supervised the project, and acquired funding. All authors contributed to the article and approved the submitted version.

## Funding

This work was supported by the National Science Foundation of China (71473152, 71774104, and 71974035), the China Medical Board (16-257), the Ministry of Education of Humanities and Social Science project (19YJCZH143), the Three-Year Initiative Plan for Strengthening Public Health System Construction in Shanghai (2020–2022) (GWV-10.1-XK23), and Cheeloo Youth Scholar Grant and Shandong University (IFYT1810 and 2012DX006).

## Conflict of Interest

The authors declare that the research was conducted in the absence of any commercial or financial relationships that could be construed as a potential conflict of interest.

## Publisher's Note

All claims expressed in this article are solely those of the authors and do not necessarily represent those of their affiliated organizations, or those of the publisher, the editors and the reviewers. Any product that may be evaluated in this article, or claim that may be made by its manufacturer, is not guaranteed or endorsed by the publisher.
