# Supplementary material for: The Effect of Parental Social Integration on the Physical Examination Utilization for Young Migrant Children: A National Cross-Sectional Study in China
Source: Front Public Health. 2022 Jan 12;9:755726. doi: 10.3389/fpubh.2021.755726 (PMC8790474; doi:10.3389/fpubh.2021.755726)
Supplement: Supplementary file 1 [file Table_1.DOCX]

Supplementary Table 1 Factor loading of the two factors of social integration (n=2,620)

|  | **Indicators** | **Factor loading** | | | |
| --- | --- | --- | --- | --- | --- |
|  |  | **Culture integration** | **Psychological integration** | | |
|  |  | **X1** | **X2** | **X3** | **X4** |
|  |  |  | **Contact intention** | **Self-**  **identity** | **Self-**  **perception** |
| 1 | Health habits are quite different from those of local citizens | **0.823** |  |  |  |
| 2 | Dressings are quite different from those of local citizens | **0.854** |  |  |  |
| 3 | Educational ideas are quite different from those of local citizens | **0.850** |  |  |  |
| 4 | Views on social issues are quite different from those of local citizens | **0.833** |  |  |  |
| 5 | Willing to live in a neighborhood with local residents |  | **0.827** |  |  |
| 6 | Willing to be colleagues with local residents |  | **0.871** |  |  |
| 7 | Willing to be neighbors with local residents |  | **0.884** |  |  |
| 8 | Willing to make friends with local residents |  | **0.867** |  |  |
| 9 | Willing to marry local residents |  | **0.606** |  |  |
| 10 | Feel that I belong to inflow areas |  |  | **0.838** |  |
| 11 | Feel that I am a member of the inflow areas |  |  | **0.867** |  |
| 12 | Think of yourself as part of the inflow areas |  |  | **0.846** |  |
| 13 | Willing to integrate into the community to become a member |  |  | **0.678** |  |
| 14 | Feel that local residents are willing to accept me as one of them |  |  | **0.639** |  |
| 15 | Feel that local residents are not willing to be neighbors with me |  |  |  | **0.850** |
| 16 | Feel that local residents don’t like me |  |  |  | **0.914** |
| 17 | Feel that local residents look down on me |  |  |  | **0.889** |
